# EEG Signal Complexity Is Reduced During Resting-State in Fragile X Syndrome

**DOI:** 10.3389/fpsyt.2021.716707

**Published:** 2021-11-11

**Authors:** Mélodie Proteau-Lemieux, Inga Sophia Knoth, Kristian Agbogba, Valérie Côté, Hazel Maridith Barlahan Biag, Angela John Thurman, Charles-Olivier Martin, Anne-Marie Bélanger, Cory Rosenfelt, Flora Tassone, Leonard J. Abbeduto, Sébastien Jacquemont, Randi Hagerman, François Bolduc, David Hessl, Andrea Schneider, Sarah Lippé

**Affiliations:** ^1^Department of Psychology, University of Montreal, Montreal, QC, Canada; ^2^Research Center of the Sainte-Justine University Hospital, Montreal, QC, Canada; ^3^University of California Davis Medical Investigation of Neurodevelopmental Disorders (MIND) Institute, Sacramento, CA, United States; ^4^Department of Pediatric Neurology, University of Alberta, Edmonton, AB, Canada; ^5^Department of Biochemistry and Molecular Medicine, University of California Davis School of Medicine, Sacramento, CA, United States; ^6^Department of Psychiatry and Behavioral Sciences, University of California Davis School of Medicine, Sacramento, CA, United States; ^7^Department of Pediatrics, University of Montreal, Montreal, QC, Canada; ^8^California North State University, College of Psychology, Rancho Cordova, CA, United States

**Keywords:** fragile X syndrome, hyperexcitability, EEG resting-state, signal complexity, multiscale entropy, alpha peak frequency, neurodevelopmental disorders, development

## Abstract

**Introduction:** Fragile X syndrome (FXS) is a genetic disorder caused by a mutation of the *fragile X mental retardation 1 gene* (*FMR1*). FXS is associated with neurophysiological abnormalities, including cortical hyperexcitability. Alterations in electroencephalogram (EEG) resting-state power spectral density (PSD) are well-defined in FXS and were found to be linked to neurodevelopmental delays. Whether non-linear dynamics of the brain signal are also altered remains to be studied.

**Methods:** In this study, resting-state EEG power, including alpha peak frequency (APF) and theta/beta ratio (TBR), as well as signal complexity using multi-scale entropy (MSE) were compared between 26 FXS participants (ages 5–28 years), and 77 neurotypical (NT) controls with a similar age distribution. Subsequently a replication study was carried out, comparing our cohort to 19 FXS participants independently recorded at a different site.

**Results:** PSD results confirmed the increased gamma, decreased alpha power and APF in FXS participants compared to NT controls. No alterations in TBR were found. Importantly, results revealed reduced signal complexity in FXS participants, specifically in higher scales, suggesting that altered signal complexity is sensitive to brain alterations in this population. The replication study mostly confirmed these results and suggested critical points of stagnation in the neurodevelopmental curve of FXS.

**Conclusion:** Signal complexity is a powerful feature that can be added to the electrophysiological biomarkers of brain maturation in FXS.

## Introduction

Fragile X syndrome (FXS) is an X-linked genetic disorder caused by dynamic mutations of the *fragile X mental retardation 1 gene* (*FMR1*), consequently leading to alterations, or to complete absence of the *fragile X mental retardation protein* (FMRP), its encoded protein. The main role of FMRP is to repress the translation of specific mRNAs during protein synthesis (1). Its absence leads to excessive protein synthesis (2), which is associated with impaired synaptic plasticity (3). FMRP is essential to brain development, as well as synaptic maturation and plasticity. FXS is the most common monogenetic cause of inherited intellectual disability (ID) and single gene cause of autism spectrum disorder (ASD). It is also associated with physical, behavioral, cognitive and emotional impairments. The clinical features of patients with FXS vary significantly from one individual to another, especially between men and women, due to the unaffected second X chromosome present in women.

Hyperexcitability is a core feature across FXS animal models and has been suggested to be a potential origin of various psychiatric and neurological symptoms observed in patients affected by the condition (4). Both overactivation of metabotropic glutamate receptors (mGluRs) leading to increased neuronal excitability (5, 6), as well as a compromised GABAergic system resulting in reduced inhibition (7), have been discussed as potential contributors to excitation/inhibition imbalance in FXS. Hence, the neurophysiological abnormalities found in humans support these notions of excitation/inhibition imbalance, including enhanced electrocortical responses and reduced intracortical inhibition, as measured by transcranial magnetic stimulation (8, 9). In addition, reduced levels of cAMP in FXS further interfere with neuronal connectivity and inhibitory responses (10). Alterations in cortical excitability may be linked to abnormal sensory processing in FXS patients. Studies investigating visual and auditory processing in FXS through event-related potentials (ERPs) with electroencephalogram (EEG) have shown important alterations in both modalities, characterized by increased amplitudes of sensory ERP components and reduced habituation to sensory stimuli (11–17).

Recent resting-state EEG studies have shown increased resting-state power in animal models of FXS, notably in delta and gamma frequency bands (18, 19). Studies with FXS adults (20), male adults (21) and young boys (22) obtained similar results. Gamma frequency bands are associated with high-level cognitive functions in healthy controls while performing cognitively demanding tasks (20, 23). However, perturbations in gamma oscillations during resting-state recordings have been reported in psychiatric disorders, as well as in neurodevelopmental conditions (20, 23–26). In fact, altered gamma power is thought to be associated with the cognitive deficits present in these populations, notably impaired social communication skills in FXS (20).

Resting-state EEG also showed evidence of increased theta power and decreased alpha power in FXS adults when compared to controls (20, 27). Alpha frequencies are the most dominant oscillations in adult resting-state EEG (20). Reduced alpha could be a marker of general brain dysfunction in FXS (27–29). Several alterations in resting-state EEG spectral domains have been identified in FXS, which could be reflected in specific EEG biomarkers of brain maturation and hyperexcitability. In particular, alpha peak frequency, theta/beta ratio, and signal complexity, a non-linear measure of brain dynamics, have been shown to be sensitive to atypical brain maturation and to the presence of neurodevelopmental disorders. This study aims at revealing whether these EEG biomarkers are affected in FXS.

First, alpha peak frequency (APF), namely the frequency at which maximum power occurs within the alpha band, shifts from theta to alpha during brain maturation. Importantly, it was found to be altered in many neurodevelopmental disorders, including attention deficit hyperactivity disorder (ADHD), ASD, and FXS (21, 28, 30).

Second, the theta/beta power ratio (TBR) of elevated slow theta waves and decreased fast beta waves is the most commonly known EEG biomarker for ADHD (31, 32). TBR could be affected in FXS since evidence of increased theta power has been shown (20, 27). However, how TBR is affected in FXS considering previous reports of elevated high beta/low gamma power in FXS is unclear.

Finally, complexity of the EEG signal is considered a marker of brain maturation and cognitive functioning (33), as it is known to increase with age. Moreover, its increase was found to be sensitive to specific sensory brain region maturation patterns (34). Although inconsistent, several studies with ADHD and ASD patients showed a general reduction in complexity, when compared to controls (35–37), while another study showed that people with ADHD have reduced complexity in the alpha frequency band (38). Multiscale entropy (MSE) is an ideal technique to quantitatively measure complexity, as it investigates temporal complexity of the signal at multiple time scales. Considering the presence of ADHD and ASD symptoms in FXS patients, it is expected that their signal complexity will also be reduced.

The present study aims to investigate, in a large sample of FXS patients, whether specific EEG markers of brain maturation, namely, APF, TBR, and complexity of the signal are affected in the condition. Here, we hypothesized that TBR of FXS patients would be elevated, and that alpha peak frequency would be reduced, compared to controls. We also predicted that FXS patients would show a reduction of EEG complexity. To our knowledge, this study is the first to explore TBR and EEG complexity in the FXS population. Furthermore, a replication study was carried out with an independently recorded additional FXS sample to ensure that EEG biomarkers can be replicated across different cohorts and study sites.

## Materials and Methods

### Participants

Thirty nine participants with a genetic diagnosis of FXS were recruited for the study. The diagnosis was based on molecular genetic examinations (39), and FXS was diagnosed when 200 or more repetitions of CGG were present. Twenty-six participants were able to complete at least a partial resting-state recording. Analysis was conducted with a final sample of 26 FXS participants. Seventy-eight neurotypical controls (NT) with a similar age distribution were recruited for the study. All neurotypical controls completed the EEG resting-state recording, but one had to be excluded due to insufficient artifact-free data. Analysis was conducted with 77 neurotypical controls. Table 1 provides demographic information on the final study population that was included for analysis. Table 2 describes the FXS population in more detail.

**Table 1:** Demographics of the study population.

	**FXS**	**Controls**
*N*	26	77
Males (*n*, %)	16 (61.54%)	40 (51.95%)
Females (*n*, %)	10 (38.46%)	37 (48.05%)
**Age**		
Mean ± SD	13.42 ± 6.7	11.55 ± 6.36
Range	5–28	5–30
**Non-verbal IQ**		
Mean ± SD	65.54 ± 22.84	110 ± 15.64
Range	36–123	44–113
**ABC-C**		
Composite score (mean ± SD)	33.35 ± 24.62	NA
Irritability subscale (mean ± SD)	9.73 ± 11.69	NA
Lethargy subscale (mean ± SD)	4.96 ± 4.22	NA
Stereotypy subscale (mean ± SD)	3.31 ± 2.87	NA
Hyperactivity subscale (mean ± SD)	9.31 ± 7.18	NA
Inappropriate speech subscale (mean ± SD)	3.73 ± 2.85	NA
Social avoidance subscale (mean ± SD)	2.31 ± 2.15	NA

*ABC-C, Aberrant Behavior Checklist-Community; IQ, Intellectual quotient; SD, Standard deviation*.

**Table 2:** Comorbid diagnoses and medication in the FXS population.

	**Male**	**Female**
*N*	15	11
**Comorbid diagnoses**		
ASD	8	1
Epilepsy	1	0
Intellectual disability	9	3
Learning disability	3	2
Speech/language impairments	3	0
**Medication**		
Antipsychotics	0	0
Antidepressants	3	1
Anxiolytics	1 (GABA supp.)	0
Psychostimulants	6	1
**Non-verbal IQ**		
Mean ± SD	60.2 ± 23.62	72.82 ± 20.54
Range	36–123	44–113
T-test	*t*_(23)_ = −1.48, *p* = 0.15	
**ABC-C**		
Composite score (mean ± SD)	39.13 ± 18.88	25.45 ± 29.95
*T*-test	*t*_(24)_ = 1.43, *p* = 0.167	
Irritability subscale (mean ± SD)	10.13 ± 9.91	9.18 ± 14.28
*T*-test	*t*_(24)_ = 0.2, *p* = 0.84	
Lethargy subscale (mean ± SD)	5.27 ± 3.86	4.55 ± 4.82
*T*-test	*t*_(24)_ = 0.42, *p* = 0.68	
Stereotypy subscale (mean ± SD)	4.33 ± 2.61	1.91 ± 2.7
*T*-test	*t*_(24)_ = 2.3, *p* = 0.03^*^	
Hyperactivity subscale (mean ± SD)	11.13 ± 6.21	6.82 ± 7.95
*T*-test	*t*_(24)_ = 1.56 *p* = 0.13	
Inappropriate speech subscale (mean ± SD)	5.2 ± 2.57	1.73 ± 1.85
*T*-test	*t*_(24)_ = 3.8, *p* = 0.001^*^	
Social avoidance subscale (mean ± SD)	3.07 ± 2.31	1.27 ± 1.42
*T*-test	*t*_(24)_ = 2.27, *p* = 0.03^*^	

*ABC-C, Aberrant Behavior Checklist-Community; ASD, Autism spectrum disorder; SD, Standard deviation*;

* *Statistically significant*.

FXS participants were recruited via the genetic clinics at the CHU Sainte-Justine Mother and Child University Hospital Center and at the University of Alberta, via parent associations and social media. NT controls were recruited via the NED lab's database of volunteers, posters and flyers in universities, colleges, and community centers, social media, and ads on classified websites. Exclusion criteria for the neurotypical group were histories of health-related problems potentially affecting development (e.g., complications during pregnancy and birth, brain trauma, epilepsy, neurodevelopmental disorders, psychopathology, etc.). The study protocol was reviewed and approved by the ethics committees at CHU Sainte-Justine and the University of Alberta and was carried out according to the declaration of Helsinki. Procedures were explained in detail prior to obtaining written informed consent from participants or legal caregivers and assent from participants.

### Behavioral Measures

A short cognitive assessment was carried out using Leiter-R (40) or Leiter-3 (41) brief IQ for FXS and most of the NT participants. Few NT participants underwent WPPSI-IV (42) or WISC-V (43) (depending on age) evaluation instead of the Leiter. For these participants, the fluid reasoning scale was selected to ensure comparability with the non-verbal Leiter batteries. PIQ results are summarized in Table 1. The revised version of the Aberrant Behavior Checklist for Community [ABC-C; (44)], specifically developed for the FXS population, was used. In this version, social avoidance, which is highly associated with ASD and FXS, was added as a sixth subscale. The ABC-C was completed by the caregiver to assess autistic traits in the clinical populations.

### Procedure

Pictograms and videos were used to prepare clinical and young NT participants for the EEG procedure. EEG net installation was adapted through storytelling and games to increase acceptance of the procedure. A movie was shown during net installation to increase collaboration in participants. For the resting-state recording, participants were told to relax as much as possible while moving as little as possible (“statues game”) keeping their eyes open and directed toward the screen where a fixation cross was displayed. If necessary, to increase acceptance and reduce movement artifacts, participants could watch a movie on the screen or favorite content on their tablet. As much as possible, resting EEG was recorded until a minimum of 2 min total of movement-free signals were obtained.

The EEG recording was carried out in soundproof experimental chambers in the CHU Sainte-Justine hospital and at the University of Alberta, using 128-electrode dense array EEG systems (Magstim EGI, Eugene, OR, USA). Signals were acquired and processed by G4 MacIntosh computers using NetStation Software (Version 4.5.4 at CHU Sainte-Justine and Version 2.0 at University of Alberta). EEG data were digitized and processed at a sampling rate of 1,000 Hz using the vertex electrode (Cz) as an online reference and an online bandpass filter of 0.1–500 Hz (Nyquist frequency) was applied. Impedances were verified prior to recording and kept below 40 kΩ (45).

### Replication Study

An additional 20 EEG resting-state datasets recorded in FXS participants were provided by the University of California Davis MIND Institute with the goal of verifying if our results can be replicated in a different cohort of FXS participants. The study was approved by the Institutional Review Board at University of California, Davis. All participants and parents/caretakers of participants gave their written consent to participate in the study. Two min of open eyes resting-state were recorded analogous to the procedure in the Montreal/Edmonton cohort. In compliant participants, alternating blocks of eyes open and eyes closed resting-state were performed. For the purpose of the current paper, only open eyes resting-state was analyzed. One participant had to be excluded since not enough clean epochs were available. Thus, 19 datasets were submitted for analysis. EEG data were acquired using a Brain Products (Brain Products, Germany) Quickamp system with an Acticap 32-channel Ag+/Ag+Cl-active electrode array according to the 10–20 international channel location system and using Brain Recorder software. EEG was digitized and processed at a sampling rate of 1,000 Hz using FCz as an online reference and an online bandpass filter of 0.1–500 Hz. Impedances were maintained below 10kΩ.

### EEG Signal Processing

#### Pre-processing

Offline analyses were carried out using MATLAB (version R2018b) and EEGLAB toolbox (v.14.1.2) (46, 47). Data were filtered with a 0.5 Hz high-pass filter, a 150 Hz low-pass filter, and a 60 Hz notch filter. For all participants recorded with the EGI 128-channel system, 28 electrodes around the face and neck were removed due to poorer signal quality in these areas. The remaining noisy electrodes were removed using a semi-automatic procedure: electrodes with a total standard deviation of >200 μV and <2 μV were automatically removed; electrodes with sporadic behavior were removed manually during subsequent visual inspection. Then, data were re-referenced to the average reference and blinks, saccades and cardiac activity were removed using independent component analysis (ICA). Continuous data was segmented into 2 s epochs using a 2-s sliding window in 1 s steps (50% overlap). This allowed us to increase the availability of clean data segments and it is also necessary for window corrections pre-PSD analyses. Artifact rejection was performed semi-automatically: epochs containing amplitudes >200 μV and < -200 μV were tagged and artifacted segments were manually removed during subsequent visual inspection, accounting for all remaining artifacts (movement etc.). Data analysis and quality metrics are presented in Supplementary Table 1. For data reduction purposes, eight regions of interest (ROI) were defined covering the following areas as closely matched as possible between EGI 128-channel and Brain Products 32-channels ActiCap locations (EGI and Brain Products): fronto-central (FCz, 5/3 electrodes for EGI/Brain Products system respectively), central (Cz, 5/3 electrodes), centro-occipital (Oz, 7/3 electrodes), /parieto-zentral (Pz, 6/4 electrodes), frontal-left (FL, 6/2 electrodes), frontal right (FR, 6/2 electrodes), temporal left (TL, 6/2 electrodes), temporal right (TR, 6/2 electrodes). Due to electrode removal during pre-processing, some participants missed some of the ROI. These were treated as missing data in subsequent analyses.

#### Power Spectral Density

Power spectral density (PSD) describes the signal distribution in terms of power per frequency using Fast Fourier Transformation (FFT). In order to reduce windowing effects, a hamming window was applied on the previously overlapped epochs before computing the FFT transform. The current method allowed us to analyze frequencies between 1 Hz and 100 Hz with a resolution of 0.5 Hz.

#### Multi-Scale Entropy

MSE was used to measure signal complexity in participants' EEG while at rest. MSE calculations were based on the algorithm proposed by (48) which generates multiple timescales through downsampling of the original EEG signal in a so-called coarse-graining procedure. The original timescale is divided into non-overlapping windows that are then averaged together. The time series shortens as window length increases. In the current study, the coarse-graining procedure was performed on all “clean” 2,000 ms epochs of resting-state data for every participant and ROI. SampEn estimates signal variability for every time series through the predictability of amplitude patterns within the time series (49). Pattern length was set to *m* = 2, meaning that the algorithm counts the number of matching sequences for two consecutive points in the signal. Tolerability was set to *r* = 0.5 indicating that amplitude points falling ≤ 50% of the time-series standard deviation equal were considered by the algorithm. Subsequently, the number of m + 1 sequences of data point matches is counted and SampEn is defined as the natural logarithm of the ratio of total *m* to *m* + 1 data point matches. Finally, MSE values for all epochs were averaged for each participant and ROI to obtain a final MSE score for every time scale from 1 to 40.

### Statistical Analysis

Statistical analyses were performed using SPSS Statistics, version 23 (IBM Corp., Armonk, NY, USA). Data distribution was verified using histograms as well as skewness/kurtosis criteria (values within−1 and 1 were considered acceptable) and z-scores. The significance level for statistical tests was set to 5% (*p* = 0.05) and Greenhouse-Geisser correction was applied to all mixed design ANOVAs. Significant interactions were investigated using follow-up ANOVAs and *post hoc* comparisons using Bonferroni correction. For PSD analysis, explorative *t*-tests between groups (FXS vs. controls) were performed for each frequency in 0.5Hz increments from 1 to 50 and 70 to 100Hz (50–70Hz were not included in the analysis due to the applied Notch filter) across all ROI in order to define frequency bands of interest. Subsequently, frequency bands of interest were averaged for each participant and ROI and compared between groups using a mixed-design ANOVA with age as covariable when appropriate. Alpha peak frequencies (APF) were defined as the frequency with maximum amplitude between 4.5 and 14 Hz for each participant and ROI. Mixed design ANOVA was carried out to compare APF between groups with age as covariable when appropriate. Theta-beta ratio (TBR) was calculated as the average of frequencies 4–8 Hz divided by the average of frequencies 14–30 Hz for each participant and ROI. TBR was compared between groups using mixed-design ANOVA with age as covariable when appropriate. For MSE, the complexity index was calculated as area-under-the-curve for scales 1–40 in order to obtain a general indication of signal complexity. Mixed design ANOVA was used to carry out group comparisons across ROIs for CI with age as covariable when appropriate. In a follow-up analysis, scales 1–20 and 21–40 were averaged in order to obtain a more fine-grained picture of differences in signal complexity between groups that were assessed in a subsequent mixed design ANOVA. Additionally, sex differences within the FXS group in APF, TBR, CI and averaged scales were assessed using mixed design ANOVA with age as covariable when appropriate. In order to assess the relationship between EEG measures and clinical outcomes, IQ and ABC-C composite score and subscales were correlated with APF, TBR, CI and averaged scales. Significance levels for correlations were corrected for multiple testing using Bonferroni's adjustment. Given that EEG measures correlated between ROI, Bonferroni's adjustment was corrected for correlated outcome variables (50). For the replication part of the study, all PSD and MSE measures were first compared between FXS cohorts using mixed design ANOVA. In subsequent exploratory analyses, the FXS replication cohort was compared to the control group to verify if results obtained in the original group comparison could be replicated. The same procedure of analyses will be followed as in the original FXS vs. controls comparison, but results will be reported with a focus on group effects to facilitate readability. Correlations between age and PSD/MSE measures were repeated in the UC Davis cohort.

## Results

We first analysed PSD in order to verify if we can replicate the results previously reported in the literature. We then analysed MSE in our cohort of FXS and control participants. Finally, we carried out a replication study with an independent sample of FXS participants to verify if our results can be replicated across cohorts and sites using different EEG systems.

### PSD

**Figure 1:**
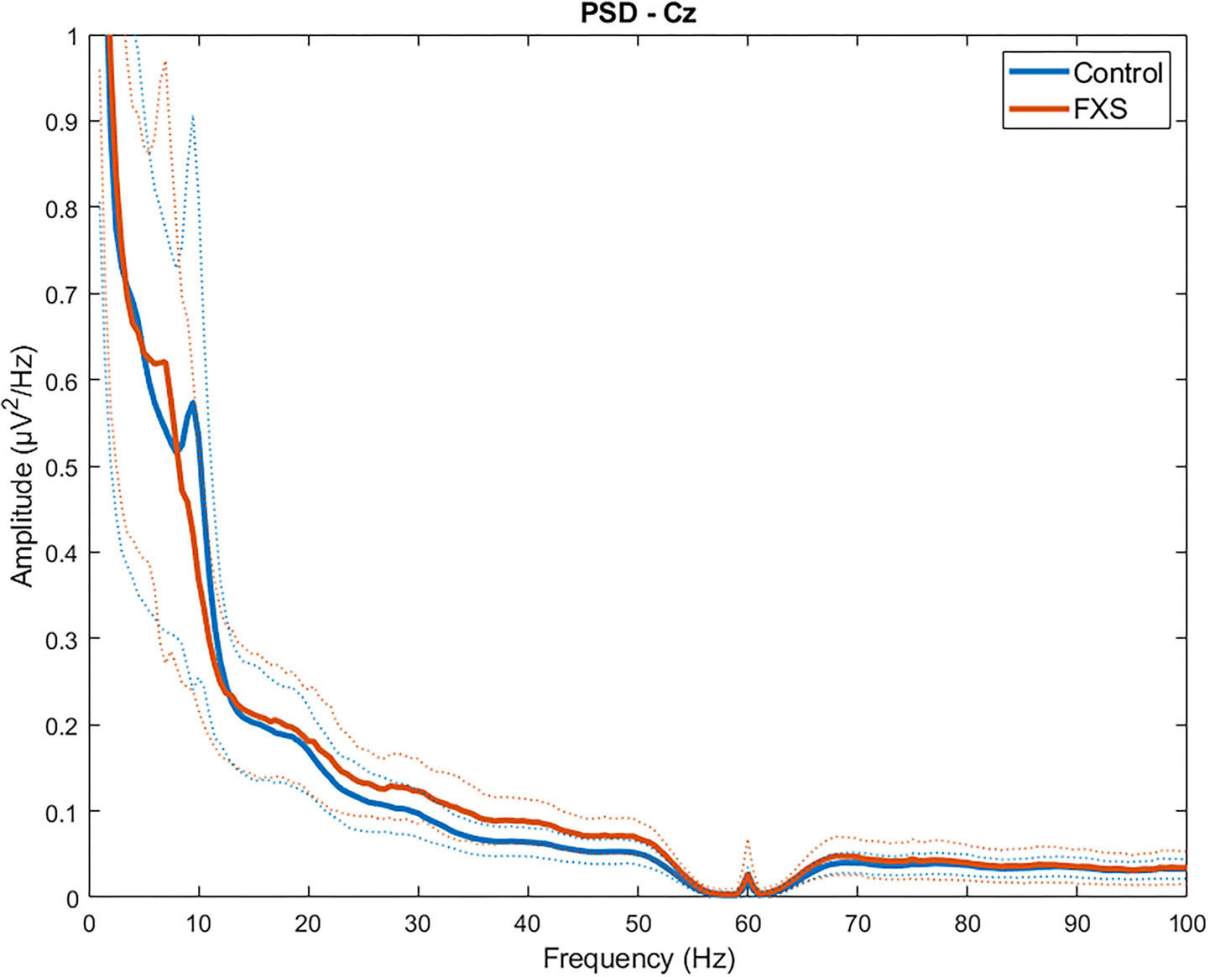
Group average power spectra for FXS vs. controls in Cz region of interest. Dotted lines indicate SD for each group.

Figure 1 shows group mean power spectra for FXS vs. controls in Cz. Explorative *t*-tests between groups (FXS vs. controls) across all ROIs revealed the following frequency bands of interest: 1–2.5 (delta) in Cz, FL, FR, FCz, TR, TL; 9.5–11 (alpha) and 25–49.5 (low gamma) in all ROI. A mixed design ANOVA controlled by age as some of the frequency bands correlated with age (*p* < 0.02), revealed significant main effects for ROI [*F*_(4.4,392.9)_ = 43.65, *p* < 0.0001, η^2^ = 0.33] frequency bands [*F*_(1.5,135.7)_ = 466.14, *p* < 0.0001, η^2^ = 0.84] and age [*F*_(1,89)_ = 6044, *p* < 0.0001, η^2^ = 0.4] and significant interactions for ROI and age [*F*_(4.4,392.9)_ = 5.88, *p* < 0.0001, η^2^ = 0.06], ROI and group [*F*_(4.4,392.9)_ = 3.66, *p* = 0.005, η^2^ = 0.04], frequency band and age [*F*_(1.5,135.6)_ = 87.7, *p* < 0.0001, η^2^ = 0.5], frequency band and group [*F*_(1.5,135.6)_ = 12.57, *p* < 0.0001, η^2^ = 0.12], ROI and frequency band [*F*_(5.6,501.4)_ = 41.19, *p* < 0.0001, η^2^ = 0.32], ROI and frequency band and age [*F*_(5.6,501.4)_ = 12.61, *p* < 0.0001, η^2^ = 0.12], ROI and frequency band and group [*F*_(5.6,501.4)_ = 4.6, *p* < 0.0001, η^2^ = 0.049]. In order to disentangle these interactions, follow-up ANOVAs per frequency band were carried out.

#### Delta (1-2.5)

**Figure 2:**
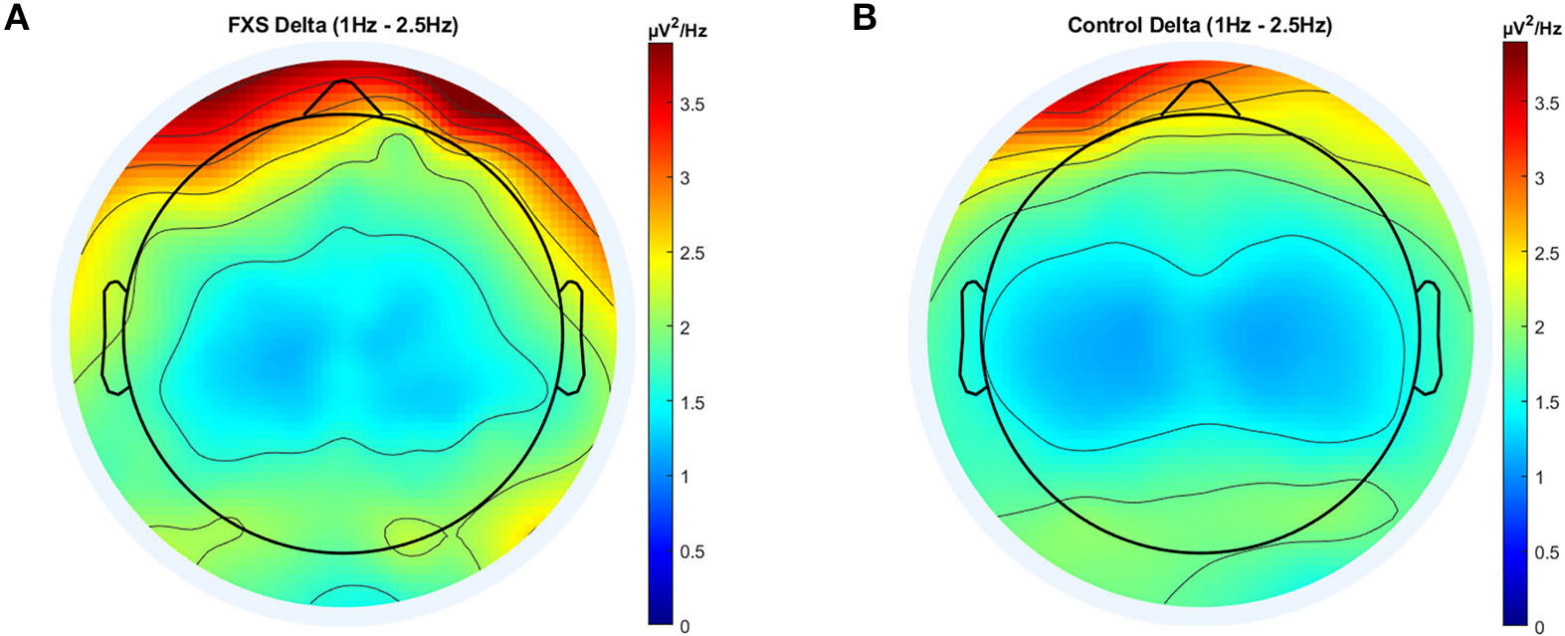
Topographic representation of average power spectral density in the FXS **(A)** and control **(B)** group for the delta band (1–2.5 Hz).

In delta, significant main effects for group [*F*_(1,97)_ = 8.14, *p* = 0.005, η^2^ = 0.07], ROI [*F*_(4.5,433.5)_ = 53.55, *p* < 0.0001, η^2^ = 0.35] and age [*F*_(1,97)_ = 100.78, *p* < 0.0001, η^2^ = 0.5] were observed as well as significant interactions between ROI and age [*F*_(4.7,433.5)_ = 11.82, *p* < 0.0001, η^2^ = 0.11] and ROI and group [*F*_(4.5,433.5)_ = 4.77, *p* = 0.001, η^2^ = 0.047]. *Post-hoc* comparisons using Bonferroni-correction revealed higher delta power in FXS (*p* = 0.005) and significant differences in delta power between almost all ROI. Figure 2 shows a topographic representation of delta power in FXS (A) and controls (B).

#### Alpha (9.5-11)

**Figure 3:**
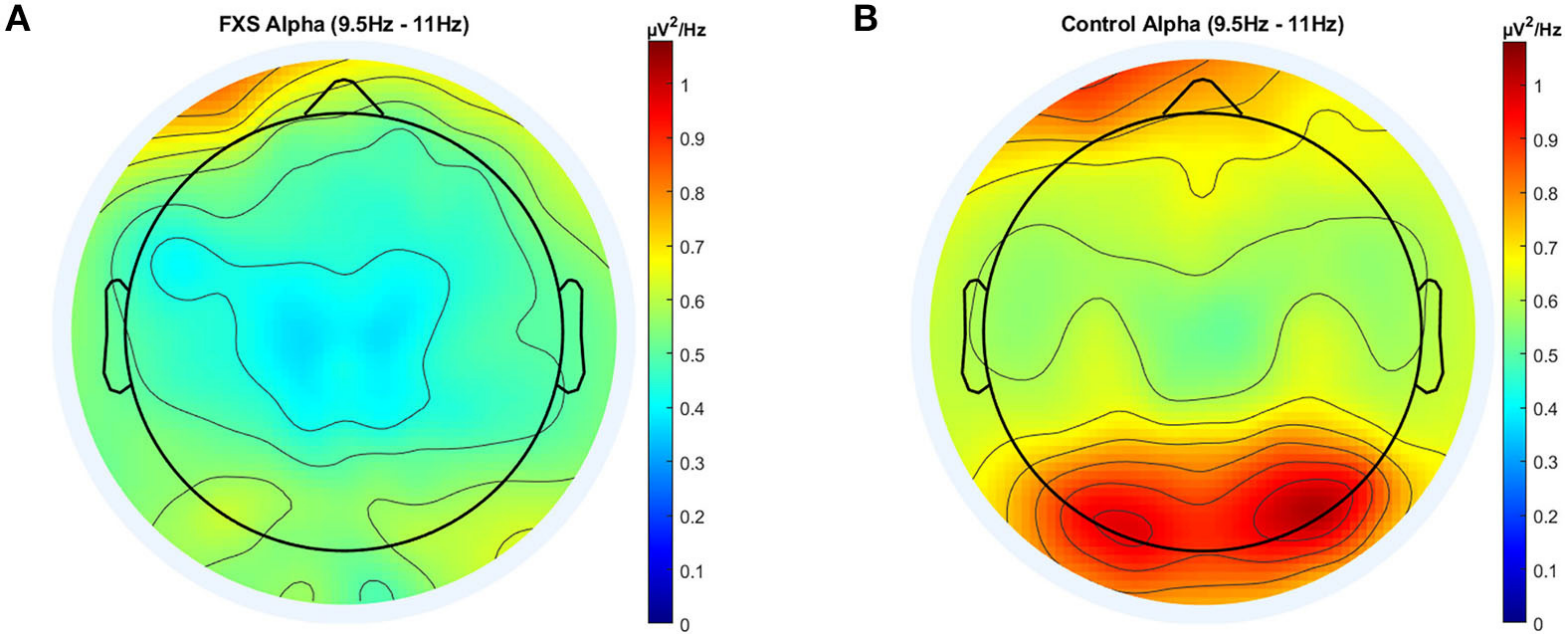
Topographic representation of average power spectral density in the FXS **(A)** and control **(B)** group for the alpha band (9.5–11 Hz).

In alpha, a significant main effect for group [*F*_(1,95)_ = 10.67, *p* = 0.002, η^2^ = 0.1] and ROI [*F*_(3.4,320.4)_ = 37.2, *p* < 0.0001, η^2^ = 0.28] was found as well as a significant interaction between ROI and group [*F*_(3.4,320.4)_ = 3.36, *p* = 0.015, η^2^ = 0.03]. *Post-hoc* comparisons using Bonferroni-correction revealed lower alpha power in FXS (*p* = 0.002) and significant differences in alpha power mostly between Cz, Oz and all other ROIs. A topographic representation of alpha power in FXS and controls is shown in Figure 3.

#### Low Gamma (25-49.5)

**Figure 4:**
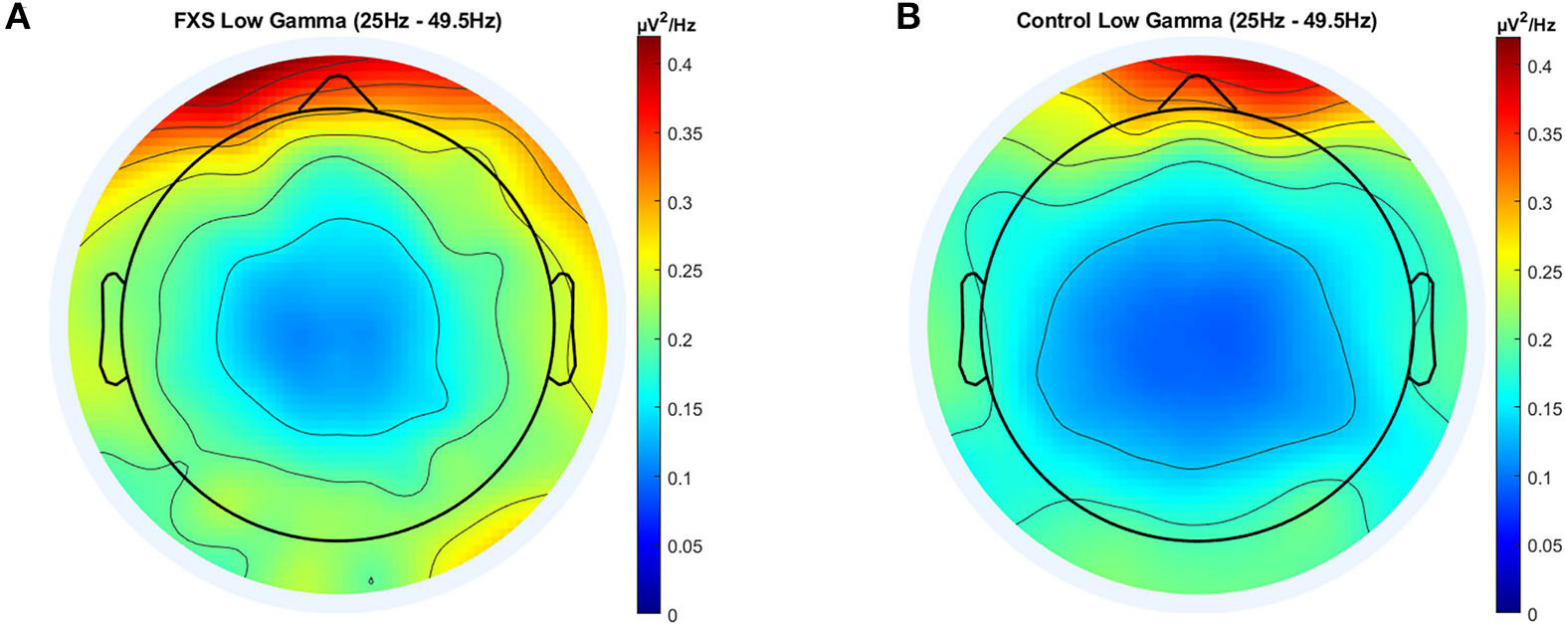
Topographic representation of average power spectral density in the FXS **(A)** and control **(B)** group for the low gamma band (25–49.5 Hz).

Significant main effects for group [*F*_(1,95)_ = 27.8, *p* < 0.0001, η^2^ = 0.23], ROI [*F*_(3.4,320.5)_ = 41.79, *p* < 0.0001, η^2^= 0.31] and age [*F*_(1,95)_ = 12.8, *p* < 0.0001, η^2^ = 0.12) were observed as well as significant interactions between ROI and age [*F*_(3.4,320.5)_ = 3.92, *p* = 0.007, η^2^ = 0.04] and ROI and group [*F*_(3.4,320.5)_ = 3.37, *p* = 0.015, η^2^ = 0.03]. *Post-hoc* comparisons using Bonferroni-correction revealed higher gamma power in FXS (*p* < 0.0001) and significant differences in gamma power between almost all ROI (*p* < 0.018). Gamma power in FXS and controls is illustrated in Figure 4.

#### Alpha Peak Frequency

**Figure 5:**
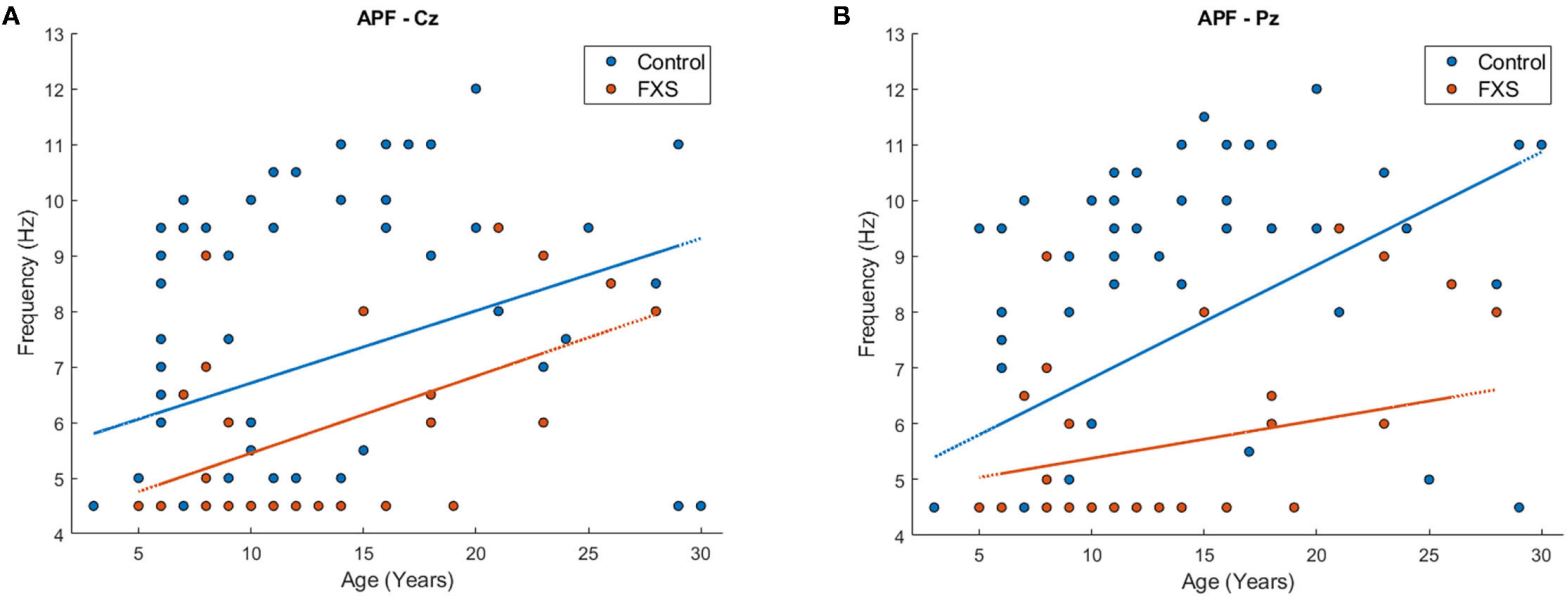
Scatterplot for age and APF for FXS (red) and controls (blue) in region of interest Cz **(A)** and Pz **(B)**.

Bonferroni's adjustment for eight correlations (one per ROI), corrected for the mean correlation between outcome variables (*r* = 0.64, *p* < 0.0001), determined a significance level of *p* < 0.024 for a correlation to be considered as significant. APF correlated positively with age in all ROI (*p* < 0.03), except Oz (*r* = 0.22, *p* = 0.024) and TR (*r* = 0.33, *p* = 0.03), confirming that APF increases with age. However, when correlations were carried out separately for FXS and controls, APF was still highly correlated with APF in controls across all ROI (*p* < 0.009); in FXS however, APF was only correlated with age in Cz (*r* = 0.54, *p* = 0.005) and FCz (*r* = 0.44, *p* = 0.023). To ensure that this effect is not simply due to the smaller sample size in the FXS group, this correlation will be repeated with the replication cohort. Figure 5 shows an exemplary scatterplot for APF and age in Cz and Pz for FXS and controls. A mixed design ANOVA [ROI (8) X group (2)] controlled for age revealed a significant main effect for group [*F*_(1,97)_ = 25.83, *p* < 0.0001, η^2^ = 0.2] and age [*F*_(1,97)_ = 27.6, *p* < 0.001, η^2^ = 0.22], indicating lower APF in FXS when compared to controls (*p* < 0.0001). No significant interactions were found.

#### Theta-Beta Ratio

For correlations, a significance level of *p* < 0.036 was determined using Bonferroni-correction adjusted for mean correlation between outcome variables (*r* = 0.85, *p* < 0.0001). TBR correlated negatively with age across all ROI in the whole sample (*p* < 0.0001) and within both groups (FXS; *p* < 0.01, controls: *p* < 0.0001). A mixed design ANOVA controlled by age revealed main effects for ROI [*F*_(4.9,439.2)_ = 41.89, *p* < 0.0001, η^2^ = 0.32] and age [*F*_(1,89)_ = 53.65, *p* < 0.0001, η^2^ = 0.38], and a significant interaction between age and ROI [*F*_(4.9,439.2)_ = 10.31, *p* < 0.0001, η^2^ = 0.1] but no group effects or interactions.

### MSE

#### Complexity Index

**Figure 6:**
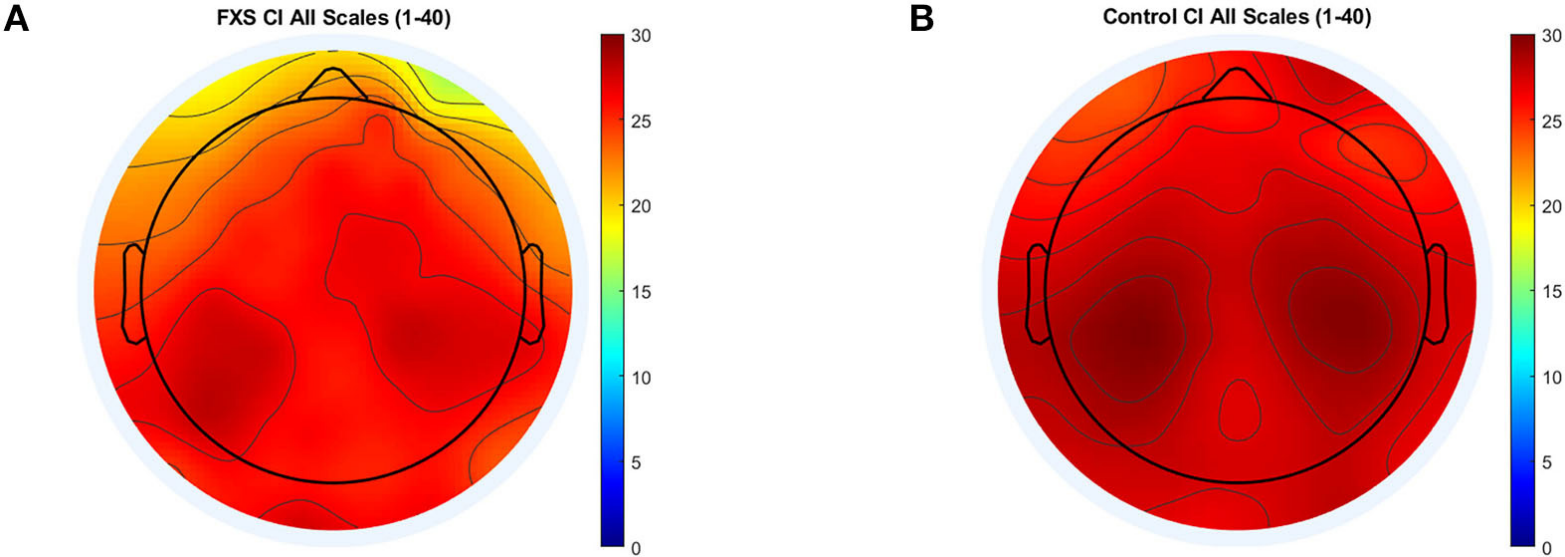
Topographic representation of complexity index (area under the curve) for MSE scales 1–40 for FXS **(A)** and controls **(B)**.

Significance level for correlations was corrected to *p* < 0.031 [Bonferroni-correction adjusted for mean correlation between outcome variables (*r* = 0.77, *p* < 0.0001)]. CI correlated highly with age across all ROI in the whole sample (*p* < 0.006). The same was found within the control group; strong positive correlations between CI and age across all ROI (*p* < 0.0001). In the FXS group however, the correlation between age and CI was less prominent and only found in central (*p* < 0.023) but not in lateral ROIs (*p* > 0.4). This correlation will be repeated in the replication study in order to verify the robustness of the result. A mixed design ANOVA controlled for age revealed main effects for ROI [*F*_(4.1,341.1)_ = 16.1, *p* < 0.0001, η^2^ = 0.16], age [*F*_(1,84)_ = 35.1, *p* < 0.0001, η^2^ = 0.3] and group [*F*_(1,84)_ = 6.45, *p* = 0.013, η^2^ = 0.07], as well as interactions between ROI and age [*F*_(4.1,341.1)_ = 8.17, *p* < 0.0001, η^2^ = 0.09] and ROI and group [*F*_(4,341.1)_ = 2.64, *p* = 0.033, η^2^ = 0.03]. Bonferroni-corrected *post hoc* comparisons revealed lower CI in FXS as compared to controls and differences between most ROI. Figure 6 shows a topographic representation of CI in FXS (A) and controls (B).

#### Averaged Time Scales S1–20, S21–40

**Figure 7:**
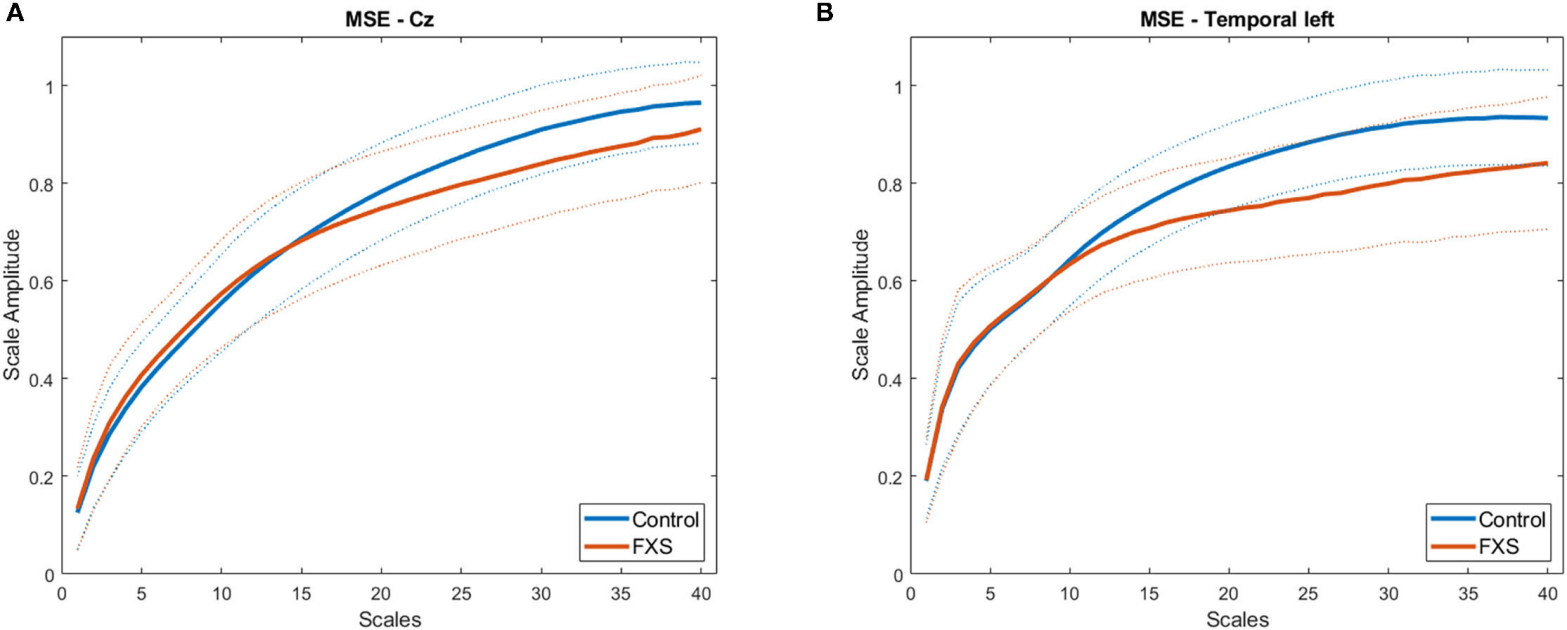
Average MSE time scales for FXS (red) and controls (blue) in regions of interest Cz **(A)** and TL **(B)**. Dotted lines indicate SD for each group.

**Figure 8:**
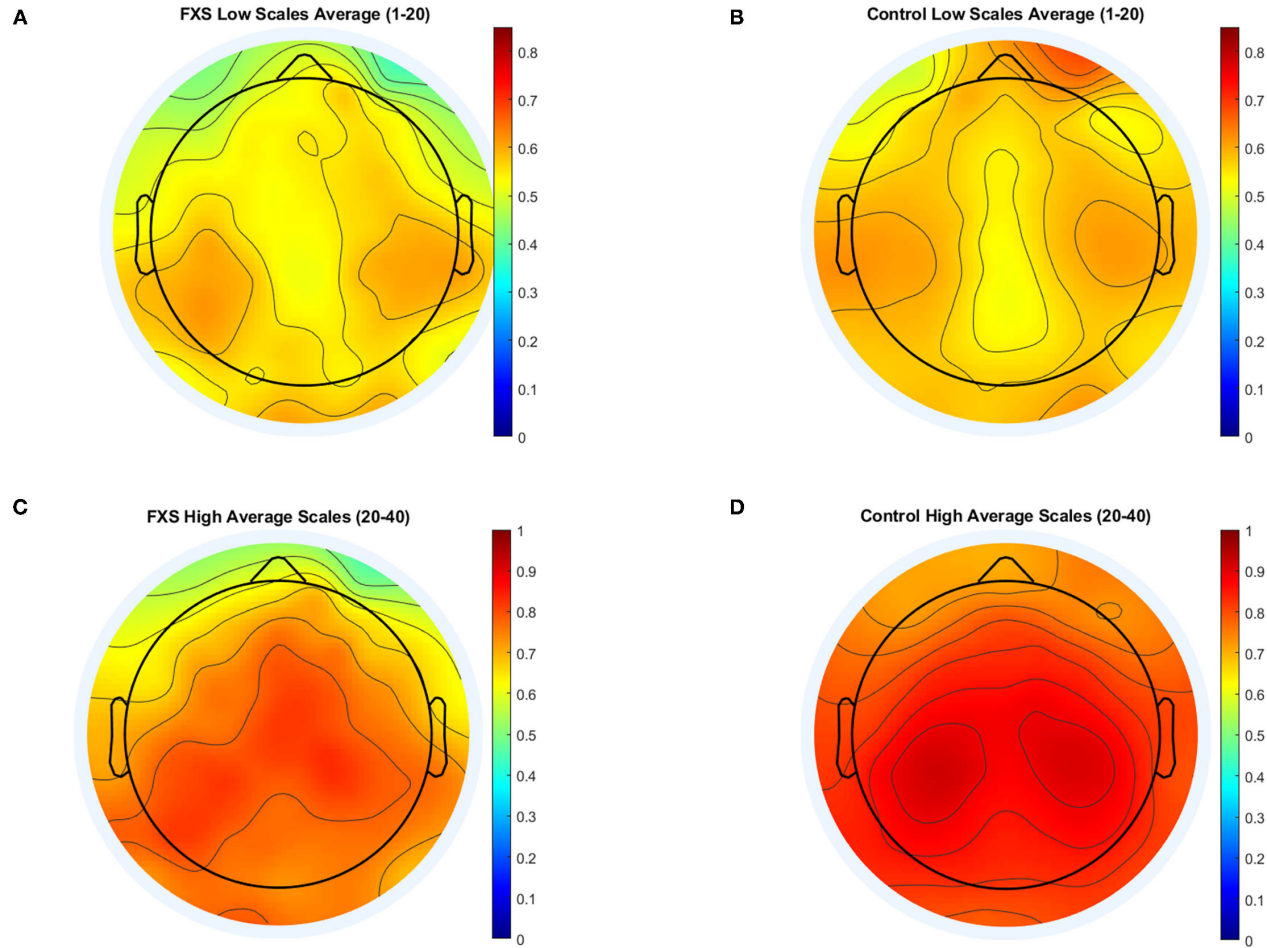
Topographic representation of averaged time scales: lower time scales (1–20) for FXS **(A)** and controls **(B)** and higher time scales for FXS **(C)** and controls **(D)**.

Figure 7 illustrates MSE across time scales for FXS and control participants in Cz (A) and TL (B). Alpha level was corrected to *p* < 0.016 adjusted for mean correlation of output variables (*r* = 0.60, *p* < 0.0001). S1–20 correlated positively with age in all ROIs (*p* < 0.0001). In controls, age correlated with S1–20 in all ROI (*p* < 0.014) and with S21–40 in all ROI except TL (*p* = 0.014). In FXS, only S1–20 correlated with age for midline ROI (Cz: *r* = 0.752, *p* < 0.0001, FCz: *r* = 0.67, *p* < 0.0001, Oz: 0.77, *p* < 0.0001, Pz: 0.69, *p* < 0.0001), whereas S21–40 did not correlate with age in FXS. A mixed design ANOVA controlled for age revealed main effects for group [*F*_(1,84)_ = 6.53, *p* = 0.012, η^2^ = 0.072], age [*F*_(1,84)_ = 34.61, *p* = 0.0001, η^2^ = 0.29], averaged scales [*F*_(1,84)_ = 335.78, *p* < 0.0001, η^2^ =0.8] and ROI [*F*_(4.1,340.7)_ = 15.96, *p* < 0.0001, η^2^ = 0.16], as well as interactions for ROI and age [*F*_(4.1,340.7)_ = 8.07, *p* < 0.0001, η^2^ = 0.09], ROI and group [*F*_(4.1,340.7)_ = 2.63, *p* = 0.034, η^2^ = 0.03], scales and age [*F*_(1,84)_ = 6.83, *p* = 0.011, η^2^ = 0.075], scales and group [*F*_(1,84)_ = 11.64, *p* = 0.001, η^2^ = 0.12], as well as ROI and scales [*F*_(4.7,393.5)_ = 12.66, *p* < 0.0001, η^2^ = 0.13]. A follow-up mixed design ANOVA per averaged scale revealed a main effect for ROI [*F*_(4.5,376.2)_ = 17.72, *p* < 0.0001, η^2^ = 0.17] and age [*F*_(1,84)_ = 60.48, *p* < 0.0001, η^2^ = 0.42] but no main effects or interactions for group in the S1–20 scales. Conversely, a mixed design ANOVA for S21–40 revealed a main group effect [*F*_(1,84)_ = 11.84, *p* = 0.001, η^2^ = 0.12] and a ROI and group interaction [*F*_(4.1,341.9)_ = 2.64, *p* = 0.033, η^2^ = 0.03], as well as main effects for ROI [*F*_(4.1,341.9)_ = 13.39, *p* < 0.0001, η^2^ = 0.14], age [*F*_(1,84)_ = 10, *p* = 0.002, η^2^ = 0.12] and an ROI and age interaction [*F*_(4.1,341.9)_ = 7.58, *p* < 0.0001, η^2^ = 0.08]. MSE was found to be lower in FXS compared to controls in higher time scales (S21–40), but not in lower time scales (S1–20). Figure 8 shows a topographic representation of S1–20 and S21–40 in FXS (A, C) and controls (B, D).

### EEG Measures and Clinical Outcomes

#### IQ

Using the Bonferroni-adjusted and for correlation between outcome variables adjusted alpha level of *p* < 0.024, APF correlated positively with IQ in the whole sample in Oz (*r* = 0.23, *p* = 0.022) and TL (*r* = 0.24, *p* = 0.016). Within groups, APF did not correlate with IQ in controls, but correlated negatively with IQ in FXS in Cz (*r* = −0.49, *p* = 0.01), FCz (*r* = −0.45, *p* = 0.023), TR (*r* = −0.51, *p* = 0.012) and TL (*r* = −0.47, *p* = 0.017). No correlations between TBR and IQ were found in the whole sample or within groups. CI, S1–20 and S20–40 were not found to be correlated with IQ for the whole sample or within groups.

#### ABC-C

Since the ABC-C is not an appropriate measurement tool for control populations, correlations were only carried out in the FXS group. APF at FL correlated positively with the inappropriate language subscale (*r* = 0.463, *p* = 0.017, note that alpha level for APF was corrected to *p* < 0.024), suggesting that FXS participants with a higher APF presented more inappropriate speech according to the ABC-C questionnaire. Further, APF in TR was found to be negatively correlated with the lethargy scale (*r* = −0.48, *p* = 0.015), suggesting less lethargy symptoms in FXS individuals with a higher APF. Expectedly, TBR correlated with the hyperactivity subscale in all frontal ROI (FL: *r* = 0.55443, *p* = 0.0063; FR: *r* = 0.51, *p* = 0.01; FCz: *r* = 0.581, *p* = 0.003; note that alpha level for TBR was corrected to *p* < 0.036), indicating that FXS participants with higher TBR presented more hyperactivity symptoms. CI correlated negatively with the ABC-C composite score at Pz (*r* = −0.56, *p* = 0.007, note that alpha level for CI was corrected to *p* < 0.031) and Cz (*r* = −0.59, *p* = 0.002); with the irritability subscale at Pz (*r* = −0.55, *p* = 0.008), TL (*r* = −0.5, *p* = 0.012) and Cz (*r* = −0.66, *p* < 0.0001); with the lethargy subscale at Pz (*r* = −0.51, *p* = 0.016); and with the hyperactivity subscale at Pz (*r* = −0.57, *p* = 0.006) and Cz (*r* = −0.61, *p* = 0.001). Similarly, S1–20 correlated negatively with the ABC composite score at Cz (*r* = −0.49, *p* = 0.011, note that alpha level for S1–20 and S21–40 was corrected to *p* < 0.016), FCz (*r* = −0.53, *p* = 0.007); with the irritability subscale at Cz (*r* = −0.57, *p* = 0.002) and FCz (*r* = −0.54, *p* = 0.007); with the hyperactivity scale at Cz (*r* = −0.62, *p* = 0.001), FCz (*r* = −0.63, *p* = 0.001) and Pz (*r* = −0.57, *p* = 0.006). Finally, S21-40 correlated negatively with the ABC-C composite score at Cz (*r* = −0.57, *p* = 0.002), FCz (*r* = −0.54, *p* = 0.006) and Pz (*r* = −0.55, *p* = 0.009); with the irritability scale at Cz (*r* = −0.63, *p* = 0.001); with the lethargy scale at FCz (*r* = −0.5, *p* = 0.01) and Pz (*r* = −0.57, *p* = 0.006); with the hyperactivity scale at Cz (*r* = −0.5, *p* = 0.01); and with the social avoidance scale at FCz (*r* = −0.6, *p* = 0.001). These correlations indicate that higher EEG signal complexity across measures predicts lower scores on the ABC-C questionnaire, specifically lower reported symptoms of irritability, lethargy, hyperactivity and social avoidance.

#### Sex Effects in the FXS Sample

Male and female FXS participants did not differ in IQ, but male participants scored significantly higher in the ABC-C composite score and some of the subscales (see Table 2 for test statistics). No sex difference was found in APF [*F*_(1,22)_ = 2.1, *p* = 0.16, η^2^ = 0.09] or TBR [*F*_(1,18)_ = 1.8, *p* = 0.2, η^2^ = 0.09] across ROI. CI did not differ between male and female participants across ROIs [*F*_(1,18)_ = 1.89, *p* = 0.19, η^2^ = 0.09]. A mixed design ANOVA revealed a weak interaction between sex, ROI and scales [*F*_(3.7,66.3)_ = 2.59, *p* = 0.049, η^2^ = 0.13]. *Post hoc* comparisons revealed that MSE was higher for males in both averaged scales in TR only [*F*_(1,20)_ = 6.5, *p* = 0.02, η^2^ = 0.24].

### Replication Study

#### Cohort Description

Table 3 contains descriptive data of the UC Davis cohort. Age did not differ between cohorts [*t*_(42.5)_ = 1.1, *p* = 0.27] but age distribution is different with the Montreal / Edmonton cohort having a peak in younger participants. Supplementary Figure 1 shows age distribution for both cohorts. Sex differed significantly between cohorts (χ*2* = 11, *p* = 0.001) since the UC Davis cohort only included one female participant. Performance IQ differed between cohorts [*t*_(42.5)_ = −2.93, *p* = 0.005], with lower IQ in the UC Davis compared to the Montreal / Edmonton cohorts. For the ABC-C measures, FXS participants differed on the inappropriate speech subscale [*t*_(41)_ = 3.29, *p* = 0.002], but no group differences were found for the ABC-C composite score or any remaining subscales (*p* > 0.51).

**Table 3:** Demographics of the “replication study cohort.”

	**FXS**
*N*	19
Males (*n*, %)	18 (94.74%)
Females (*n*, %)	1 (5.26%)
**Age**		
Mean ± SD	15.26 ± 4.32
Range	8–22
**Non-verbal IQ**		
Mean ± SD	48.86 ± 14.76
**ABC-C**		
Composite score (mean ± SD)	51.27 ± 32.02
Irritability subscale (mean ± SD)	13.44 ± 13.01
Lethargy subscale (mean ± SD)	7.41 ± 6.88
Stereotypy subscale (mean ± SD)	6.32 ± 5.91
Hyperactivity subscale (mean ± SD)	9.56 ± 6.46
Inappropriate speech subscale (mean ± SD)	6.76 ± 3.11
Social avoidance subscale (mean ± SD)	3.68 ± 3.61

*ABC-C, Aberrant Behavior Checklist-Community; FXS, Fragile X syndrome; SD, Standard deviation*.

#### PSD

APF did not correlate with age for any ROI in the UC Davis cohort (*p* > 0.08), whereas it was found to correlate positively with age in the Montreal/Edmonton cohort. TBR did not correlate with age after alpha level was Bonferroni-corrected and adjusted for the mean correlation of outcome variables (*r* = 0.82, *p* < 0.0001) to *p* < 0.034, whereas it had been found to correlate negatively with age in the Montreal/Edmonton cohort. Mixed design ANOVAs did not reveal any significant differences between cohorts in APF [*F*_(1,36)_ = 2.75, *p* = 0.11, η^2^ = 0.07] or TBR [*F*_(1,32)_ = 0.53, *p* = 0.5, η^2^ = 0.02] nor significant ROI and cohort interactions {APF: [*F*_(4.7,169.34)_ = 0.72, *p* = 0.6, η^2^ = 0.02], TBR: [*F*_(4.1,131.8)_ = 1.52, *p* = 0.24, η^2^ = 0.05]} but a main effect for ROI {APF: [*F*_(4.7,169.3)_ = 3.29, *p* = 0.009, η^2^ = 0.08], TBR: [*F*_(4.1,131.8)_ = 20.53, *p* < 0.0001, η^2^ = 0.4]}. When compared to the control group, APF and TBR effects could be replicated in the replication cohort with significantly lower APF in FXS *F*_(1,85)_ = 7.3, *p* = 0.008, η^2^ = 0.08) and no group effects or interactions for TBR *F*_(1,81)_ = 2.98, *p* = 0.088, η^2^ = 0.04). A mixed design ANOVA revealed no FXS cohort effect for delta, alpha and low gamma [*F*_(1,33)_ = 2.43, *p* = 0.13, η^2^ = 0.07], but a significant ROI, cohort and frequency band interaction [*F*_(4.1,135.8)_ = 5.45, *p* = 0.0001, η^2^ = 0.14], as well as main effects for ROI [*F*_(4.6,150.3)_ = 49.5, *p* < 0.001, η^2^ = 0.6], frequency bands [*F*_(1.2,38.5)_ = 251.37, *p* < 0.0001, η^2^ = 0.87] and a ROI and frequency band interaction [*F*_(4.1,135.8)_ = 25.38, *p* < 0.0001, η^2^ = 0.44]. Follow up ANOVAs by ROI revealed a cohort main effect [*F*_(1,38)_ = 7.94, *p* = 0.008, η^2^ = 0.17] in Oz only. A significant interaction between frequency bands and groups [FXS replication cohort vs. controls: *F*_(1.7,132.5)_ = 21.55, *p* < 0.0001, η^2^ = 0.84] in a mixed design age-controlled ANOVA covering delta, alpha and low gamma frequency bands led to follow-up analysis by frequency band. Compared to controls, the replication FXS cohort presented higher delta [*F*_(1,86)_ = 27, *p* < 0.0001, η^2^ = 0.24] and gamma power *F*_(1,85)_ = 95.8, *p* < 0.0001, η^2^= 0.53), whereas no group effects were found in alpha power [*F*_(1,82)_ = 0.007, *p* = 0.93, η^2^ < 0.0001].

#### MSE

CI, S1–20 and S21–40 did not correlate with age in any ROI for the UC Davis cohort (*p* > 1.82), whereas a slight correlation between CI and S1–20 and age was found in central ROI in the Montreal/Edmonton cohort. A mixed design ANOVA revealed no cohort main effect for CI [*F*_(1,31)_ = 0.015, *p* = 0.9, η^2^ = 0.0004], or averaged MSE scales [*F*_(1,31)_ = 0.017, *p* = 0.9, η^2^ = 0.001] nor any cohort and ROI interactions {CI: [*F*_(2.5,76.4)_ =2.47, *p* = 0.08, η^2^ = 0.074], averaged scales: [*F*_(2.5,76.4)_ = 2.47, *p* = 0.08, η^2^ = 0.074]} or averaged scales and cohort interaction [*F*_(1,31)_ = 0.79, *p* = 0.38, η^2^ = 0.025]. A ROI main effect was found for both CI [*F*_(2.5,76.4)_ = 4.66, *p* = 0.008, η^2^ = 0.13] and averaged scales [*F*_(2.3,76.4)_ = 4.6, *p* < 0.0001, η^2^ = 0.13]. When compared to controls, the FXS replication cohort presented a lower CI [*F*_(1,77)_ = 5.8, *p* < 0.018, η^2^ = 0.07] as well as lower complexity in S21–40 [*F*_(1,77)_ = 10.86, *p* = 0.001, η^2^ = 0.12], whereas no group differences were found in S1–20 [*F*_(1,77)_ = 0.08, *p* < 0.78, η^2^ = 0.001].

#### Clinical Outcome Measures

IQ did not correlate with APF in the replication cohort, whereas negative correlations between IQ and APF in several ROI occurred in our original FXS sample. Further, no correlations were found between IQ and TBR, CI, S1–20 or S21–40 in our replication cohort, thus replicating the results of our original FXS sample. Analyses with the ABC-C scores were not performed in the replication cohort since missing data did not allow for a sufficient N to carry out correlations.

## Discussion

Several EEG markers were previously found relevant to brain maturation and hyperexcitability in the FXS population. The results of the present study replicated these findings. APF and alpha power were found to be decreased, and gamma power was increased in the FXS groups, compared to controls. Furthermore, this study was the first to show reduced signal complexity in higher time scales, as well as increased delta power in FXS participants. The main results of our study are summarized in Table 4.

**Table 4:** Main results of the FXS cohorts.

	**FXS vs. controls**	**FXS replication cohort vs. controls**	**FXS vs. FXS replication cohort**
**Power spectral density**			
Delta	Higher delta power in FXS	Higher delta power in FXS	**Only in Oz**: slightly higher PSD in replication cohort, no other significant differences
Alpha	Lower alpha power in FXS	No significant difference	
Low gamma	Higher gamma power in FXS	Higher gamma power in FXS	
Alpha peak frequency	Lower alpha peak frequency in FXS	Lower alpha peak frequency in FXS	No significant difference
Theta-beta ratio	No significant difference	No significant difference	No significant difference
**Multiscale entropy**			
Complexity index	Lower CI in FXS	Lower CI in FXS	No significant difference
Average time scales (S1–20)	No significant difference	No significant difference	No significant difference
Average time scales (S21–40)	Lower MSE in FXS	Lower MSE in FXS	No significant difference

*CI, Complexity index; FXS, Fragile X syndrome; MSE, Multiscale entropy*.

### MSE

EEG signal complexity was found to be significantly reduced in FXS participants, in both cohorts, compared to healthy controls. A decrease in signal complexity is concordant with alterations in brain maturation and developmental delay characterizing individuals with FXS. Diminished EEG signal complexity in FXS participants was found in all regions of interest investigated, suggesting it is a global phenomenon across resting-state signal generators.

Several studies found a general increase in brain signal complexity with age in neurotypical populations. Indeed, studies have found neurodevelopmental effects on signal complexity from infancy through adolescence (34, 51, 52). This developmental increase was clearly observed in our control group across all regions of interest. In FXS it is however a lot less evident, as CI only correlates with age in central and fronto-central regions and no age-related increase was found in the remaining regions of interest. This discrepancy could be explained by the smaller sample size of the FXS cohort. Moreover, in our FXS replication cohort, where the peak of the age distribution is found toward the end of the teenage years rather than in childhood, and that contains lower functioning individuals, the complexity index does not at all correlate with age. These results support the growing discrepancy with age found in FXS compared to neurotypical children, as a certain stagnation in CI development seems to take place during the teenage years. Our cohorts, ranging from 5 to 28 years old, allowed us to show this discrepancy. Our results suggest that early school years are when trajectories of signal complexity maturation differ across brain regions in FXS. However, whether EEG signal complexity differences are present before 5 years of age remains to be studied.

Some studies identified signal complexity as a relevant EEG marker of neurodevelopmental disorders. These studies also found a general reduction in EEG signal complexity in ADHD, ASD, and Tourette Syndrome (35–37, 53), as well as reduced complexity in the alpha frequency band (38). MSE could potentially be a useful biomarker in establishing the neurodevelopmental and neurobehavioral trajectories of patients with FXS. Augmented complexity was found to be correlated with lower ABC-C composite scores, as well as fewer symptoms on the irritability, lethargy, hyperactivity, and social avoidance subscales. A recent study investigating behavioral characteristics found in different clinical populations reported that patients with FXS scored higher on the irritability, lethargy and hyperactivity subscales than NT controls (54). These results are consistent with our observations and suggest that signal complexity might be reflecting the behavioral impairments associated with FXS. Since we have not excluded any comorbidities in our FXS population, those comorbidities could also have an impact on the reduced complexity found.

Reduced signal complexity in FXS was expected as several previous studies found unitary/simplified brain processes in this population. Topographically, Knoth et al. (14) found that a lower number of spatial principal components could explain FXS brain signals. Moreover, Côté et al. (11, 13), found low variability between trials of sensory responses, together with high amplitudes, suggesting a potential for synchronization of brain signals (55). Increased phase synchronization to sensory stimuli was indeed found in low-frequency bands (<20 Hz) in FXS (56). Notably, the significant reduction in MSE values of FXS is found in the higher/coarser scales, potentially capturing lower frequency oscillations, while the finer-grained scales, where the increase in low gamma power observed in our FXS cohort could have introduced variability or noise, were not found significantly different.

### PSD et TBR

The FXS group showed higher delta power, lower alpha power, and increased low gamma power. Results of the replication study cohort also showed higher delta power and increased low gamma power. These results were expected as they confirm previous studies, supporting a robust signature of FXS resting-state EEG (20–22, 27). Whereas, TBR was found to stabilize toward less discrepancies between theta and beta power with age, it did not differ between FXS and controls. In fact, 5–10 Hz frequency power is seemingly reduced, whereas high beta and low gamma powers are increased. Hence, although not significant, the TBR is potentially flattened by the modifications in spectral power found in FXS. Several medications, including the psychostimulant types, are known to modify beta power (57). Whether the absence of results is due to medication taken by the FXS participants remains to be studied. Despite the fact that TBR is considered an electrophysiological biomarker in the ADHD population, what TBR reflects is still a matter of debate (32, 58). Here, we found a positive relationship between TBR in the left frontal area of interest and the hyperactivity subscale of the ABC-C. Those results are consistent with the literature reporting an association between hyperactivity and higher TBR in ADHD children (59). A recent study found that resting-state TBR is not altered in ADHD but is positively correlated with the inattentive symptoms of the disorder (60). Since the ADHD-inattentive sub-type is prevalent in the FXS population (61), hypotheses of interaction between TBR and inattention will be worth exploring in future research.

Alpha peak frequency is a long-standing EEG marker of brain maturation (62). During development, APF migrates from the theta frequency range to the alpha frequency range. APF was indeed associated with age in our neurotypical control group. In the FXS group, correlations were found only in central but not lateral regions of interest—comparable to our results found for the relationship between CI and age in FXS. Again, no correlation between age and APF was found in our replication cohort that is characterized by a later peak in age distribution and lower functioning individuals. Considering that at an early age theta is particularly robust in central regions, these results are consistent with the growing discrepancies in brain function with age in FXS, and support the evidence showing a failure to shift the APF with age in children with ASD (63). Importantly, APF was significantly lower in both FXS cohorts, ranging in the theta frequency range, rather than the alpha frequency range. Although APF has not been widely investigated in FXS, reduced alpha power has been reported by several authors, in humans (20, 27, 64) and in rats (65). Our results are also consistent with studies of neurodevelopmental disorders showing reduced alpha power in children with ASD (30, 63) and ADHD (28), and decreased APF in children with ASD (62). Increased APF was also associated with higher scores on the inappropriate speech subscale. An association between APF and language acquisition has been reported in the literature (66), suggesting that FXS participants who scored higher on the inappropriate speech subscale might have better language abilities in general, though their speech may be unsuitable for certain situations. Notably, alpha power and APF were not found altered in female FXS patients in previous studies (21, 64). However, we did not find any sex differences in APF in our FXS sample. This discrepancy is potentially due to the fact that our FXS population is more diversified in terms of cognitive functioning, in both the male and female FXS participants.

### Mechanisms Behind the Scenes

Hence, several EEG markers are seemingly characteristic of the FXS brain. They may be mechanistically divided into two categories of indices: delay of maturation, and hyperexcitability. Increase in delta power, reduced alpha peak frequency, and diminished EEG signal complexity are EEG markers that have been associated repeatedly with brain maturation (34, 62, 67–69). FXS individuals carrying the full mutation show a flattened curve of cognitive neurodevelopment with a plateau around 6 years of age. Decades of research on the function of FMRP identified several mechanisms underlying FXS. Through the FMRP role in translational control, long-term synaptic and spine morphological plasticity, FXS is genuinely a neurodevelopmental disorder. FMRP is developmentally regulated, at least in mice, and implicated in the experience-dependent plasticity mechanisms of neurodevelopment, of which its dysregulation seemingly leads to permanent changes. Whether FMRP expression levels at specific moments during development are revealed by EEG markers most associated with brain maturation remains to be tested. Certainly, an increase in slow frequency band density, decrease in alpha power and alterations in signal complexity have been associated with several neurodevelopmental disorders affecting cognitive and behavioral neurodevelopment. In children with ASD, signal complexity was sensitive to the severity level of the symptoms (70). Notably, the significant differences changed topographically according to age from 4 to 8 years old. In our study, significant interactions between age and regions of interest were found in spectral power, complexity index and higher MSE time scales. Hence, the most group difference-sensitive EEG indices may vary during the course of development. Acquisition of data from younger FXS participants could enable the identification of specific critical moments during development where the neurodevelopmental trajectories diverge, thereby identifying ultimate periods to administer treatments for maximal gain.

On the other hand, increases in gamma power and reductions in alpha power have been associated with hyperexcitability in the FXS population. In a cognitive neuroscience framework, the alpha and gamma frequency bands have functional interactions, where alpha pulses are inhibited, reducing processing capacities in a given brain area. In this framework, alpha power is reduced by attention, and gamma oscillations are increased to process information (71). This inhibitory process, which happens through alpha activity (72), is thought to be driven by GABAergic interneurons (27). In the context of FXS, the impaired alpha activity could be generated by altered gamma power. Indeed, gamma activity is directly modulated by inhibitory GABAergic interneurons (65). The GABAergic system is known to be altered in FXS (7), consequently leading to hyperexcitability and increased gamma power. Furthermore, alpha oscillations reflect a neural mechanism aiming to gate the processing of external sensory information, altered in FXS (20, 27), as well as in other conditions such as pain (73).

From a neuroscience perspective, it has been established that local circuit glutamate-GABA interactions are part of the neural mechanisms underlying gamma activity (55), and that these interactions are altered in FXS. Indeed, overactivation of glutamatergic circuits and hypoactivation of GABAergic circuits have been documented (74). Furthermore, local circuit inhibitory interneurons are known to play an important role in regulating the flow of excitatory networks by providing inhibitory control (75). However, fast spiking inhibitory GABAergic interneurons, which are involved in high-frequency neural activity, are dysfunctional (12, 19, 56), leading to reduced local inhibition, and consequently resulting in neuronal hyperexcitability. Thus, our results support the gamma activity abnormalities and contribute to a better understanding of the cortical excitation/inhibition imbalance found in FXS (12, 65).

## Conclusion

Our study confirms that several EEG markers characterizing brain maturation and hyperexcitability are altered in FXS. Moreover, the results obtained with our replication study cohort showed that results using different EEG systems are replicable, not only between FXS cohorts, but also between a FXS cohort and healthy controls. These results are encouraging and suggest the feasibility of multi-site studies. Future studies should consider pooling data using normalization techniques. Although further studies in younger participants are required, our results suggest critical points of stagnation in the neurodevelopmental curve that can be assessed particularly by signal complexity and alpha peak frequency, as they have been shown to be sensitive to both brain maturation and FXS phenotype. Hence, the significant findings obtained on MSE, APF, as well as delta, alpha, and gamma power suggest that several EEG atypicalities could be used as biomarkers for FXS. The next step is to determine if these markers are responsive to pharmacological treatments targeting specific mechanisms.

## Data Availability Statement

The original contributions presented in the study are included in the article/Supplementary Material, further inquiries can be directed to the corresponding author/s.

## Ethics Statement

The studies involving human participants were reviewed and approved by Ethics Committees at CHU Sainte-Justine, the University of Alberta, and the University of California, Davis. Written informed consent to participate in this study was provided by the participants' legal guardian/next of kin.

## Author Contributions

MP-L and IK performed most of the analyses and wrote the manuscript. KA performed several analyses and created the figures. VC, HB, AT, C-OM, A-MB, and CR tested the participants and contributed to EEG signal analyses. Co-researchers FT, LA, SJ, RH, FB, DH, and AS contributed to the scientific conception of the study. SL is the senior scientist, she conceived the EEG project, and contributed to analyses, interpretation and writing. All authors contributed to the article and approved the submitted version.

## Funding

This research was funded by the Azrieli Foundation, the Canadian Institutes of Health Research (CIHR) (grant number 142346) and the Natural Sciences and Engineering Research Council of Canada NSERC (grant number 386207).

## Conflict of Interest

The authors declare that the research was conducted in the absence of any commercial or financial relationships that could be construed as a potential conflict of interest.

## Publisher's Note

All claims expressed in this article are solely those of the authors and do not necessarily represent those of their affiliated organizations, or those of the publisher, the editors and the reviewers. Any product that may be evaluated in this article, or claim that may be made by its manufacturer, is not guaranteed or endorsed by the publisher.
