# Corrigendum: EEG Signal Complexity Is Reduced During Resting-State in Fragile X Syndrome

**DOI:** 10.3389/fpsyt.2022.867000

**Published:** 2022-02-24

**Authors:** Mélodie Proteau-Lemieux, Inga Sophia Knoth, Kristian Agbogba, Valérie Côté, Hazel Maridith Barlahan Biag, Angela John Thurman, Charles-Olivier Martin, Anne-Marie Bélanger, Cory Rosenfelt, Flora Tassone, Leonard J. Abbeduto, Sébastien Jacquemont, Randi Hagerman, François Bolduc, David Hessl, Andrea Schneider, Sarah Lippé

**Affiliations:** ^1^Department of Psychology, University of Montreal, Montreal, QC, Canada; ^2^Research Center of the Sainte-Justine University Hospital, Montreal, QC, Canada; ^3^University of California Davis Medical Investigation of Neurodevelopmental Disorders (MIND) Institute, Sacramento, CA, United States; ^4^Department of Pediatric Neurology, University of Alberta, Edmonton, AB, Canada; ^5^Department of Biochemistry and Molecular Medicine, University of California Davis School of Medicine, Sacramento, CA, United States; ^6^Department of Psychiatry and Behavioral Sciences, University of California Davis School of Medicine, Sacramento, CA, United States; ^7^Department of Pediatrics, University of Montreal, Montreal, QC, Canada; ^8^California North State University, College of Psychology, Rancho Cordova, CA, United States

**Keywords:** fragile X syndrome, hyperexcitability, EEG resting-state, signal complexity, multiscale entropy, alpha peak frequency, neurodevelopmental disorders, development

In the original article, there was an error. The abstract states that we compared 26 FXS participants with 7 neurotypical controls. This is incorrect. As correctly stated in the methods and result sections, we compared 26 FXS participants to 77 neurotypical controls.

A correction has been made to **Methods** section of the **Abstract**.

**Methods:** In this study, resting-state EEG power, including alpha peak frequency (APF) and theta/beta ratio (TBR), as well as signal complexity using multi-scale entropy (MSE) were compared between 26 FXS participants (ages 5–28 years), and 77 neurotypical (NT) controls with a similar age distribution. Subsequently a replication study was carried out, comparing our cohort to 19 FXS participants independently recorded at a different site.

The authors apologize for this error and state that this does not change the scientific conclusions of the article in any way. The original article has been updated.

## Publisher's Note

All claims expressed in this article are solely those of the authors and do not necessarily represent those of their affiliated organizations, or those of the publisher, the editors and the reviewers. Any product that may be evaluated in this article, or claim that may be made by its manufacturer, is not guaranteed or endorsed by the publisher.

