# Supplementary material for: EEG Signal Complexity Is Reduced During Resting-State in Fragile X Syndrome
Source: Front Psychiatry. 2021 Nov 11;12:716707. doi: 10.3389/fpsyt.2021.716707 (PMC8632368; doi:10.3389/fpsyt.2021.716707)
Supplement: Supplementary file 1 [file Table_1.docx]

**Table 1. Pre-processing parameters**

|  | FXS | Controls | FXS replication cohort |
| --- | --- | --- | --- |
| Number of ICA components removed  Mean±SD | 1.42±0.83 | 2±1 | 0.82±1.12 |
| Epochs kept for analysis  Mean±SD | 148.6±66.31 | 243.76±87.83 | 103.17±40.75 |
| Epoch retention rate  Mean±SD | 54.02%±21% | 69.65%±18% | 60%±18% |

*FXS= Fragile X syndrome; ICA= Independent component analysis; SD= Standard deviation*
