# Supplementary figures and images for: EEG Signal Complexity Is Reduced During Resting-State in Fragile X Syndrome
Source: Front Psychiatry. 2021 Nov 11;12:716707. doi: 10.3389/fpsyt.2021.716707 (PMC8632368; doi:10.3389/fpsyt.2021.716707)

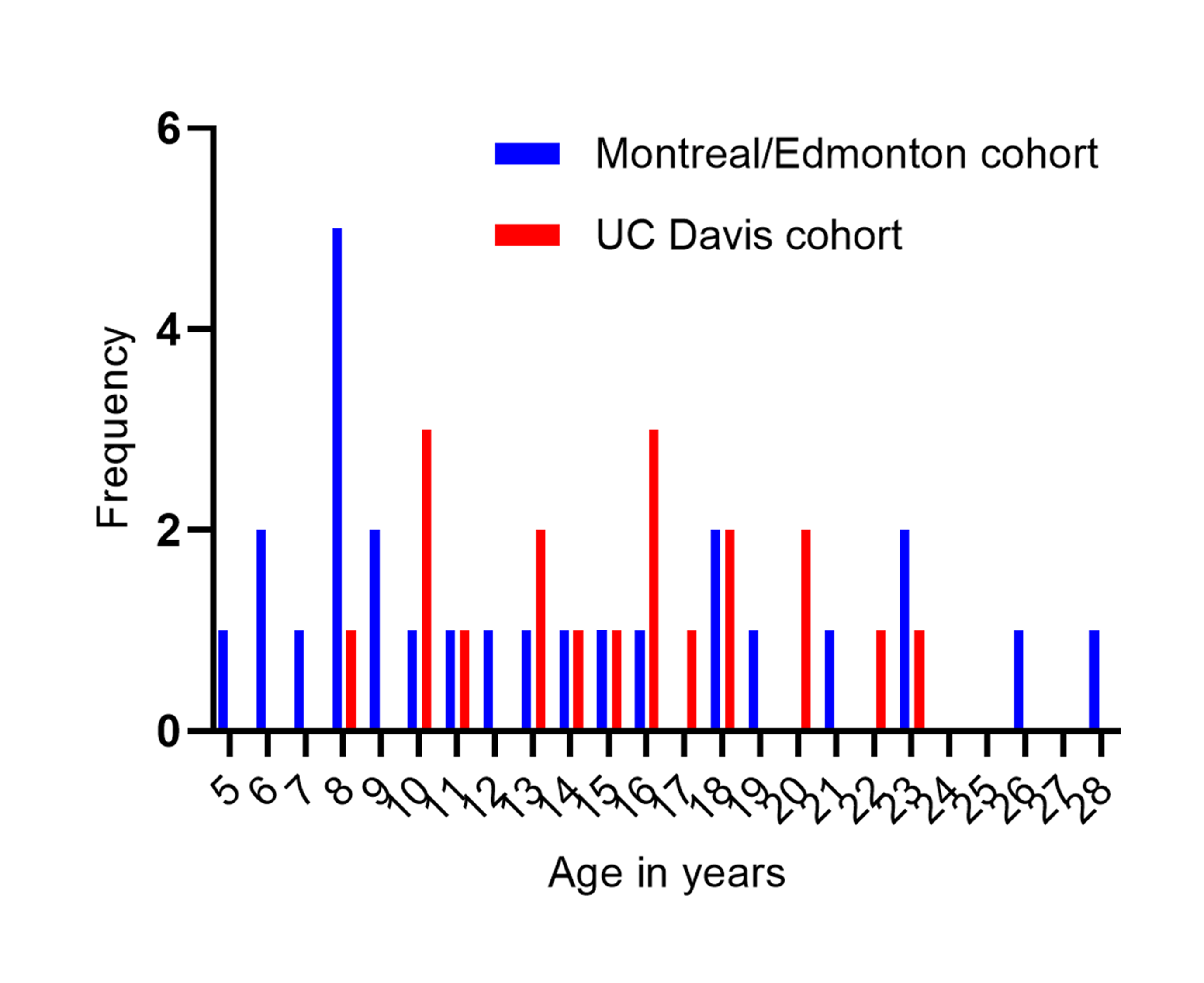

Supplement: Supplementary file 2 [file Image_1.TIF]
